# Effectiveness of a Batteryless and Wireless Wearable Sensor System for Identifying Bed and Chair Exits in Healthy Older People

**DOI:** 10.3390/s16040546

**Published:** 2016-04-15

**Authors:** Roberto Luis Shinmoto Torres, Renuka Visvanathan, Stephen Hoskins, Anton van den Hengel, Damith C. Ranasinghe

**Affiliations:** 1 Auto-ID Lab, The University of Adelaide, North Terrace, Adelaide SA 5005, Australia; damith.ranasinghe@adelaide.edu.au; 2 Aged & Extended Care Services, The Queen Elizabeth Hospital, Woodville South SA 5011, Australia; renuka.visvanathan@adelaide.edu.au (R.V.); Stephen.Hoskins@health.sa.gov.au (S.H.); 3 Adelaide Geriatrics Training and Research with Aged Care (GTRAC) Centre, The University of Adelaide, North Terrace, Adelaide SA 5005, Australia; 4 Australian Centre for Visual Technologies, The University of Adelaide, North Terrace, Adelaide SA 5005, Australia; anton.vandenhengel@adelaide.edu.au

**Keywords:** fall prevention, bed exits, chair exits, weighted conditional random fields, older people

## Abstract

Aging populations are increasing worldwide and strategies to minimize the impact of falls on older people need to be examined. Falls in hospitals are common and current hospital technological implementations use localized sensors on beds and chairs to alert caregivers of unsupervised patient ambulations; however, such systems have high false alarm rates. We investigate the recognition of bed and chair exits in real-time using a wireless wearable sensor worn by healthy older volunteers. Fourteen healthy older participants joined in supervised trials. They wore a batteryless, lightweight and wireless sensor over their attire and performed a set of broadly scripted activities. We developed a movement monitoring approach for the recognition of bed and chair exits based on a machine learning activity predictor. We investigated the effectiveness of our approach in generating bed and chair exit alerts in two possible clinical deployments (Room 1 and Room 2). The system obtained recall results above 93% (Room 2) and 94% (Room 1) for bed and chair exits, respectively. Precision was >78% and 67%, respectively, while F-score was >84% and 77% for bed and chair exits, respectively. This system has potential for real-time monitoring but further research in the final target population of older people is necessary.

## 1. Introduction

Falls occur commonly in hospitals, especially in older people with dementia or delirium, where about 30% of falls result in some type of injury [1]. Falls in hospitals have been reported in the literature to occur inside the patient’s rooms (84%) and during ambulation (19%) [1]. Moreover, the majority of falls occur around the bed and chair area [2,3]. Falls are costly as patients have a longer length of stay in hospital wards and other related expenses. The estimated cost of a fall related hospitalization in the United States of America is US$50 534 per person (inflation adjusted since 2006) [4]. Randomized controlled trials showcasing multiple component interventions have had limited success in the reduction of falls. The interventions used include patient assessments, exercise, medication or fixed bed or chair exit alarms where motions such as bed and chair exits trigger an alarm to provide caregivers an opportunity to provide assistance [5,6]. Moreover, there is no clear indication of which component contributed most to the reduction of falls.

Other clinical studies, such as that of Shorr *et al.* [7] and the recent study of Sahota *et al.* [8] focused on fall prevention using only the technological components of fixed bed and chair exit alarms. In these approaches, motions such as bed and chair exits trigger an alarm to provide caregivers an opportunity to provide assistance when such activities are being attempted without supervision. These studies reported no changes in falls after using pressure sensors. Although the performance of the sensors was not reported, a contributing factor for these results may be the high rate of false alarms leading to “alarm fatigue” in caregivers [7]. The study of Wong-Shee *et al.* [9] used similar bed and chair alarms achieving significant reduction of falls when compared to the pre-intervention period; however, there was no significant reduction of falls when compared to the post-intervention period. This study reported the number of alarms, producing as many false alarms as approximately 80% of true detected alarms. Additionally, other recent approaches such as the use of bed mats with multiple sensors [10] or side rails with pressure sensors [11] obtained high performance but were tested on young and middle-aged volunteers rather than on older participants. Moreover, it has been reported that the use of bed rails to prevent falls actually increases the risk of injury as it raises the height of a fall [12]. Multiple studies have also used video images for fall prevention; however, previous research has manifested privacy concerns with the use of cameras in older people’s living environments [13].

The use of wearable sensors provide new opportunities for monitoring patients [14,15]. However, most studies are focused on the monitoring of activities or gait [16,17,18,19], or the assessment of falls risk [20,21], which evaluates the long term risk of the person by using a self-evaluation tool and targets mostly older people living independently. Researchers have investigated sensor units on the torso consisting of an accelerometer, in some cases in combination with other sensors such as gyroscopes, magnetometers and barometers. Nonetheless, these wearable units were bulky as they were battery operated and in some cases required the patient to be wired, which is not recommended for older people. Other wearable sensor based studies used more than one sensing device attached to the participant’s body; for example, in [22], a participant wore wearable sensors (IMOTE2, (sensor node platform developed by Intel Research) and radio frequency identification (RFID) readers on both wrists and a third mote on the body, an approach that is uncomfortable for older people as they are heavily instrumented.

There is a lack of studies using wearable technology to prevent falls that are capable of notifying a caregiver [23]. A recent successful study by Wolf *et al.* [24] trialled a commercial sensor unit—Shimmer (Shimmer, Dublin, Ireland) equipped with a tri-axial accelerometer—With hospital patients for the detection of bed exits. Like previous studies, the sensing unit is expensive, relatively heavy, battery powered and needed to be strapped to the leg, which can be uncomfortable to some patients. In this study, we are interested in investigating a batteryless and lightweight wearable data collection platform that has the potential to be inconspicuous to the person wearing it, while also able to collect and transmit data from the user. Moreover, recent studies have demonstrated that older people have an interest in lightweight sensors that can be embedded in their clothes for monitoring [25], and that chest-located sensors [26] are better able to capture upper-body movements to effectively determine postures in older people [16,17].

**Figure 1:**
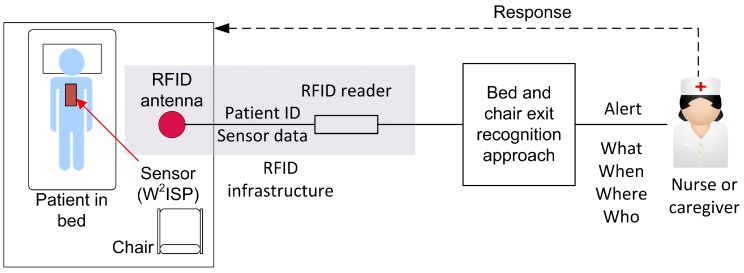
Overview of the proposed fall prevention technological intervention. Data collected by the radio frequency identification (RFID) infrastructure is sent in real-time to the bed and chair exit recognition approach stage. Caregivers can be notified via alert messages to assist the electronically identified patient (*who*) that is performing a bed or chair exit (*what*), the alert is issued in real-time (*when*) and the RFID antenna and reader identifiers can indicate the room occupied by the patient (*where*).

The present work focuses on methods for the monitoring of activities in older people using a batteryless wearable sensor which is part of a larger technological intervention to prevent falls (see Figure 1). This study builds on previous research from our team for the detection of bed exits using wearable technology; in [27], we investigated the use of an empirical algorithm on a cohort of young people. In [28], we used a batteryless sensor enabled RFID device, called W^2^ISP (Wearable Wireless Identification and Sensing Platform) [29,30], with a cohort of healthy older people where the W^2^ISP was worn over their clothing for the collection of human motion information and was found to be acceptable and non-obstructive among the cohort of older people that took part in the trials. Unlike in [28], this study focuses on the recognition of both bed and chair exits and the real-time prediction of activities from streaming sensor data. This characteristic is important for clinicians [25] as a way for them to make instant assessments, prioritize supervision as well as provide a timely intervention to prevent a fall.

The main objective of the present study is to evaluate the performance of our batteryless sensor based bed and chair exit recognition approach for real-time identification of bed and chair exits. We investigate the application of our wearable device in a population of healthy older people using two different deployment options suitable for hospitals. The bed and chair exit recognition approach consists of: (i) a feature extraction stage; (ii) an activity prediction model built based on a statistical learning method called dynamically weighted conditional random fields (dWCRF) that learns class related weight parameters during training; and (iii) a bed and chair exit recognition algorithm. In order to support future research in the area of wearable sensors for human activity recognition in older populations, we have also made the data used in this study publicly available (http://autoidlab.cs.adelaide.edu.au/research/ahr).

The rest of the paper is organized as follows. Section 2 presents the concept of our technological intervention for fall prevention; Section 3 details our trial and data processing methods. Results and discussion are described in Section 4, and Section 5 presents our conclusions and future work.

## 2. Technological Intervention

This study is part of a proposed general deployment, shown in Figure 1, that is part of a larger and ongoing intervention strategy being researched for fall prevention for hospitalized older people [31]. Our approach is based on RFID, a technology used in various healthcare applications [32,33], where passive RFID tags—Batteryless, small and inexpensive devices—Can be easily replaced or disposed to prevent possible spread of infections. Moreover, RFID platforms are increasingly being deployed in hospitals, a current reality for monitoring the location of equipment, patients and personnel [34,35]; therefore, integration with an existing RFID infrastructure (*i.e.*, RFID antennas and readers) can result in ease of integration with existing systems and the reduction of operational costs.

**Figure 2:**
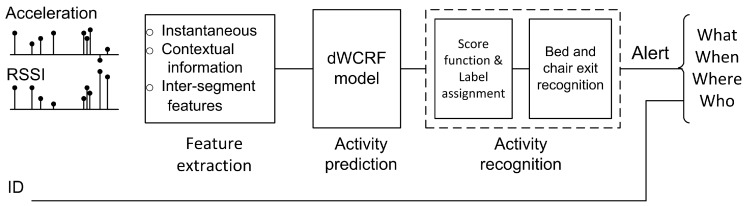
Proposed bed and chair exit recognition approach. Acceleration data from the sensor and additional information such as the received strength of the signal (RSSI) evaluated by the RFID reader are inputs to the recognition approach.

In our proposed intervention, the participants, using a batteryless wearable sensor, have data corresponding to their movements and their identification being collected in real-time via the RFID infrastructure (see Figure 1). The data is received at the bed and chair exit recognition approach stage for processing and analysis (shown in detail in Figure 2); this stage issues an alert to caregivers to assist the identified patient after a bed or chair exit has been recognized.

### 2.1. Sensor Technology

**Figure 3:**
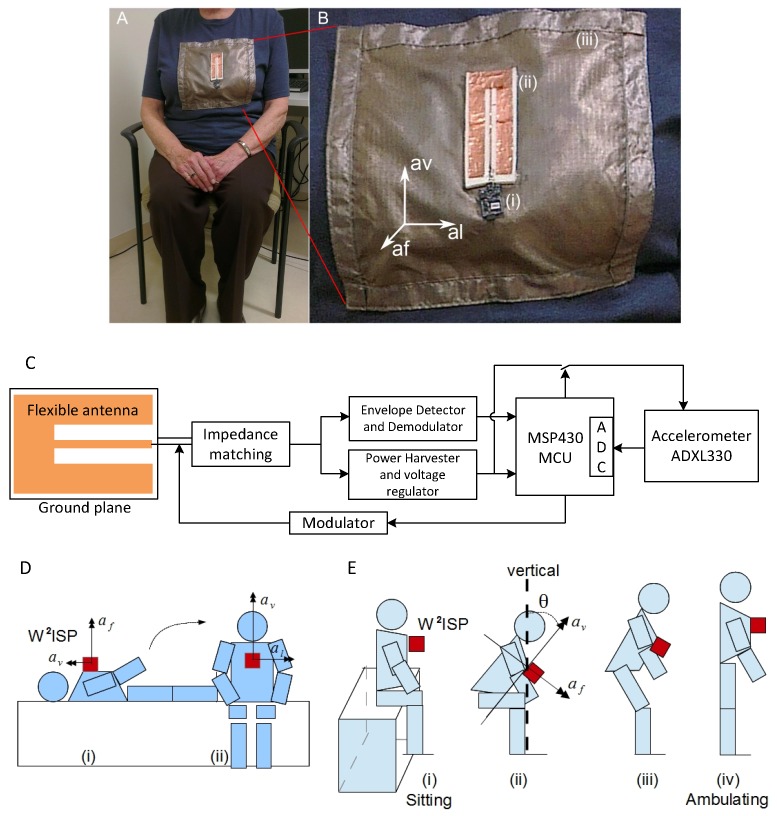
Wearable Wireless Identification and Sensing Platform (W^2^ISP). (**A**) older volunteer wearing device on top of clothing; (**B**) W^2^ISP parts: (i) circuitry, 18×20×2 mm; (ii) flexible antenna, 36×85×2 mm; and (iii) isolating silver coated fabric, 230×220 mm; (**C**) block diagram of W^2^ISP platform; (**D**) process of lying on bed to sitting on bed; and (**E**) process of sitting (in bed or chair) to ambulating.

We used a flexible Wearable Wireless Identification and Sensing Platform (W^2^ISP) device developed by our team, based on [29], suitable for wear over a garment anterior to the sternum as shown in Figure 3A [28]. The W^2^ISP is a passive RFID tag that includes an accelerometer and a microcontroller unit. The 3-axis accelerometer (ADXL330) has a minimum full scale range of ±3 g and low power requirement of 180 μA with a supply voltage of 1.8 V and typical output sensitivity of 300mV/g. The microcontroller (MSP430F2132) is a 16-bit flash, ultra low power consumption unit that also includes a 10-bit, 200 kilo-samples-per-second Analog to Digital Converter (ADC). A block diagram of the W^2^ISP platform displaying main components is shown in Figure 3C.

The W^2^ISP includes a printed circuit board based RFID circuitry module and sensing unit with a flexible antenna (referred to as sensor hereafter) for patient comfort and a washable RIPSTOP silver coated nylon fabric to isolate device and human (Figure 3) [30]. The W^2^ISP communicates with off-the-shelf UHF RFID readers and harvests its power using the electromagnetic (EM) field illuminating the tag from the RFID reader antennas. The RFID reader transmits interrogation signals to the passive tags using the 920–926 MHz ISM (industrial, scientific and medical) band under Australian electromagnetic compatibility regulations. The W^2^ISP responds with its unique ID and sensor information by backscattering and modulating the incident RF signal from the reader [36]. The strength of the received backscattered signal captured by an RFID reader antenna and processed by a reader is called received signal strength indicator (RSSI); other information relative to the RF signal such as frequency channel is also collected.

### 2.2. Bed and Chair Exit Recognition Approach

The approach shown in Figure 2 consists of three main stages: (i) feature extraction; (ii) activity prediction; and (iii) activity recognition process. Feature extraction refers to obtaining essential information from the sensor data stream from which the activity predictor can accurately infer the likelihood of the performed activity; the set of predicted activities (classes) in this study are: (i) Sitting-on-bed; (ii) Sitting-on-chair; (iii) Lying; and (iv) Ambulating. The activity recognition process collects the output of the activity predictor, assigns an activity to each sensor observation using a score function and generates an alert using the activity recognition algorithm in the event a bed or chair exit is recognized. The use of a score function is important to reduce the number of misclassification errors as discussed in Section 3.2.3.

## 3. Methods

### 3.1. Data Collection

#### 3.1.1. Study Participants

This study had ethics approval by the Human Research Ethics Committee of the Queen Elizabeth Hospital (protocol number 2011129). Fourteen volunteers participated in the study, they were 65 years and older with no cognitive impairment and able to mobilize independently. Participants were recruited from geriatric clinics or from lists of interested volunteers who had participated in other geriatric research studies. Request for participation was over the phone. Written informed consent was obtained and no honorarium was provided. During the trial, a researcher was present to instruct the participants the activities that needed to be performed from a script. The same researcher also annotated the activities in the sensor data capturing software built by the research team. Participants were informed of the activities contained in the script to ensure that they had no objections to any of the proposed activities but were not informed of the order before the trial start.

#### 3.1.2. Clinical Setting and Procedure

**Figure 4:**
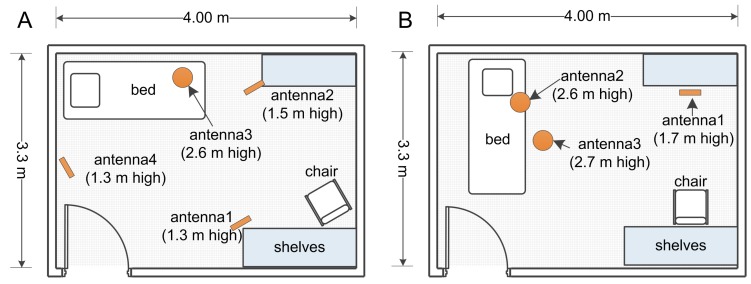
The two room configurations used in the study. Configuration of equipment with antennas on ceiling level shown as circles and vertical antennas shown as rectangles facing either the bed or chair. (**A**) Room 1, antenna3 is at ceiling level on top of the bed and the rest of the antennas are on a vertical stand. Antenna2 is inclined towards the chair and antenna1 and antenna4 face front (chest level) towards the bed area; and (**B**) Room 2, antenna2 and antenna3 are at ceiling level tilted towards the bed and antenna1 is on a vertical stand inclined towards the chair.

The study was undertaken within two different clinic room configurations: (i) Room 1, with one antenna located on a high stand at ceiling level facing down to the bed, and three other antennas located on vertical stands facing front; and (ii) Room 2, with two antennas located on high stands at ceiling level facing towards the bed area and an antenna on a vertical stand facing the chair (Figure 4). These settings were designed to closely resemble two single room configurations common in a hospital environment (single bed and arm chair in the room). Each room configuration yielded a dataset. We refer to the corresponding data set obtained from each room as Room 1 dataset and Room 2 dataset.

Each participant was assigned to one room setting and randomly allocated to undertake approximately five trials using one of two broadly scripted lists of activities of daily living that included walking to the chair, sitting on the chair, getting off the chair, walking to bed, lying on bed, getting off the bed and walking to the door. Participants were instructed at the beginning of the trial to perform each activity at their own pace and as comfortably as possible; no other instruction was given as to how to perform each activity. In general, participants performed more in-bed activities, *i.e.*, sitting and lying in bed, than sitting on the chair, and, typically, the trials included twice as many lying on bed activities as sitting on chair activities. Consequently, the participants spent more time sitting or lying than ambulating. This is also reflective of hospitalized older people where rooms are small and furniture such as chairs and bed are in close proximity and where patients spend more time on the bed than on the chair. Participants were also instructed to request a trial termination if they were distressed or in discomfort. The duration of the trials per participant was between 90 to 120 min and was performed during the day between 10 am and 3 pm. A researcher annotated in real-time the activities being undertaken (ground truth), and this was later contrasted with activities as determined by the algorithm to measure the system performance.

### 3.2. Data Processing

#### 3.2.1. Feature Extraction

This stage extracts from the sensorial data the features that describe the underlying signal patterns of body motions as input to the activity prediction stage. We consider time domain features extracted from the W^2^ISP readings such as: acceleration, time, phase, frequency channel and RSSI.

**Figure 5:**
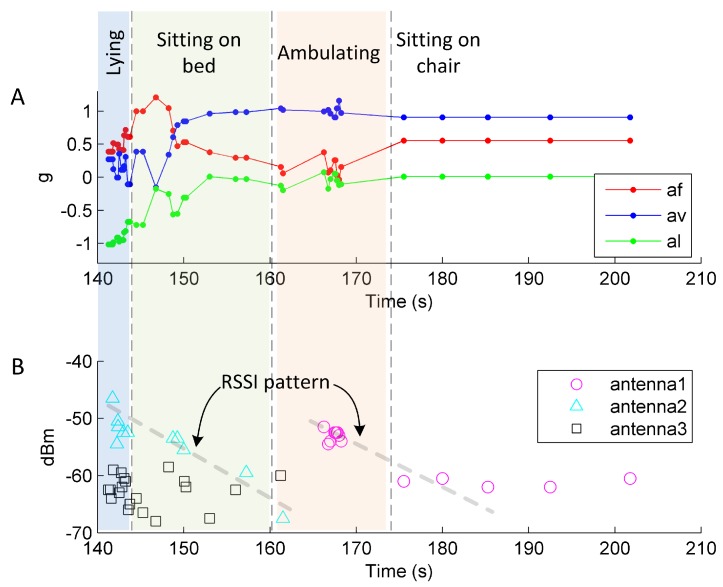
Sample of collected sensor data. (**A**) Raw accelerometer readings along the three axes; and (**B**) RSSI (received signal strength indicator) received from three antennas in the room and RSSI pattern changes across four activities.

A challenging aspect of feature extraction is the formulation of features given noisy and irregular sensor observations. A limitation of RFID technology originates in the tag powering, as it depends on EM illumination from RFID antennas to collect and transfer data from the sensor. Hence, the effects of variable distance to antenna, destructive interference due to multipath, radio frequency band interference and occlusion by RF opaque objects such as the human body cause irregular, incomplete and noisy readings that are delivered to the bed and chair exit recognition approach shown in Figure 2. We can see in Figure 5 some of these effects as incomplete signals are collected from a study participant wearing the sensor where some readings are separated by several seconds, as is the case when the participant is sitting on the chair. This irregularity can cause the loss of sensor readings during posture transitions, e.g., getting out from bed, and can cause a person to have similar sensor readings before and after changing posture. For instance, a person with the sensor on the chest is first sitting on a chair and later stands up, or *vice versa*, these two postures can potentially have similar sensor readings as the person’s body has similar chest orientation during both sitting on chair and standing.

We are interested in using acceleration data from the W^2^ISP as they contain information about the performed movement. Common features in the literature consider the use of frequency-domain features (e.g., frequency components, energy and entropy) [37], which require regularly sampled data or interpolation methods that add post-processing stages to our method. Hence, we consider time-domain features from acceleration signals and additional sources of information from the received RF signal.

We are particularly interested in RSSI as an indicator of relative proximity to a reading antenna as low values can represent the participant being further from the reading antenna than higher RSSI values. This is because RSSI (1) is inversely proportional to the direct distance ($d_{o}$) from transmitting and receiving antennas [38], given by the equation:

(1) $$\text{RSSI}=\frac{KP_{t}G_{t}\lambda^{4}{\left|H\right|}^{4}}{{\left(4\pi d_{o}\right)}^{4}}$$

where *K* is the tag backscatter gain, $P_{t}$ is the output power of the reader, $G_{t}$ is the gain of the monostatic reader antenna and *H* is the channel response to multipath [38]. Previous studies have established the importance and utility of RSSI based features. In [39], the combined use of RSSI with acceleration based features improved the classifier performance compared to using acceleration based features by themselves; this study [39] also demonstrated that the combination of features provided similar or better performance to using time and frequency domain features from acceleration readings alone. In [27], variations in RSSI data were useful to determine changes in postures that would otherwise be difficult to discriminate using only acceleration based data, e.g., a person sitting in the chair and standing has the participant’s trunk to be upright in both postures. Other information such as RF phase measures the phase angle between the RF carrier (at a given frequency channel) transmitted by the RFID reader and the returned signal from the sensor [40]. Similar to RSSI measurements, phase changes are sensitive to variations in distance and motion of the sensor [40].

Despite the irregular and noisy nature of RFID data, we can see in Figure 5A that the transfer of a person from lying to sitting on bed is evident in the acceleration readings and the RSSI patterns from the readings from antenna2 (see Figure 5B). In contrast, the transition from ambulating to sitting on chair cannot be clearly determined by using acceleration alone; however, the RSSI patterns for antenna1 can help discriminate this transition.

Therefore, we combine time domain information in RSSI, phase, frequency channel and acceleration sensor data and employ three categories of features based on studies in [16,28,39,40,41]; we describe them in detail below.

##### Instantaneous features:

These features are obtained from the current reported sensor observation and provide information about the action being performed as reported by the sensor and the participant information. However, no general information of what is occurring in the temporal vicinity is provided. Features included are:

- Accelerometer readings in the three axes: av, al and af shown in Figure 3B;
- Body tilting forwards and backwards given by $\sin\left(\theta \right)=\sin(\arctan\left(\frac{af}{av}\right))$, *θ* shown in Figure 3E [16,28];
- Rotational angle $\text{yaw}=\arctan(\frac{al}{af})$;
- Rotational angle $\text{roll}=\arctan(\frac{al}{av})$;
- ID of the antenna receiving the sensor data (aID) [28];
- Received power from the sensor (RSSI) [28];
- Time difference with the previous observation; and
- Gender of the person.

##### Contextual information features:

These features are obtained from a 4 s fixed time sliding window segment where the first element in the segment corresponds to the current sensor observation. This segmentation method was evaluated in Shinmoto Torres *et al.* [41] for extraction of contextual information returning good prediction performance and is easily implemented [41]. These features provide an insight into the temporal variations of the sensor information during the segment, hence covering the deficiencies of instantaneous features. The segment information is important as events distant in time (outside the segment) become less relevant to the current activity. Features included are:

- Importance of each antenna in collecting sensor observations given by the relative number of readings per antenna in the segment [41];
- Mutual information between bed and chair areas given by: $\frac{1}{n}\sum_{k=1}^{n-1}1_{\left[\left\{aID_{k},aID_{k+1}\right\}=\left\{chair,bed\right\}\right]}+1_{\left[\left\{aID_{k},aID_{k+1}\right\}=\left\{bed,chair\right\}\right]}$, where $1_{x}$ is the indicator function and *n* the number of elements in the segment [41];
- IDs of antennas receiving maximum and minimum RSSI in segments;
- Displacement in the av axis (Figure 3B), given by: ${\;\int \int \;}_{t1-4s}^{t1}av\;dt^{2}$;
- Mean${}^{†}$ and standard deviation${}^{†}$ of acceleration readings av, al and af;
- Mean${}^{†}$ and standard deviation${}^{†}$ of RSSI for all antennas;
- Pearson correlation between pairs of acceleration axes;
- Standard deviation${}^{†}$ of variable frequency phase rate (VFPR) [40]; and
- Sum of modulus${}^{†}$ of constant frequency phase rate (CFPR) [40].

##### Inter-segment features:

These features are obtained from the differences in information from consecutive segments. These features characterize the acceleration and received signal power variation trends over successive segments. These pattern variation trends provide information about motion and relative proximity to the area of interest, *i.e.*, bed or chair, that is not affected by noise. The inter-segment information trends are obtained using the features:

- Difference of median, maximum and minimum of acceleration readings av, al and af from consecutive segments; and
- Difference of median, maximum and minimum of RSSI per antenna from consecutive segments.

We also performed feature selection using the data from each room configuration, Room 1 and Room 2, before introducing the data to the Activity predictor stage. We selected simple classifiers such as random forests and probabilistic models such as Bayes network and logistic regression, eliminating low ranked features. Hence, the features above are used for both room configurations, except those of the form $f^{†}$ which are used by Room 1 alone.

#### 3.2.2. Activity Predictor

The activity prediction stage is based on the probabilistic modeling method of linear chain conditional random fields (CRF) [42], a structured classifier that models the dependencies of activities in a sequence, *i.e.*, takes into consideration the information from adjacent observations rather than considering each observation as independent of each other. Moreover, our data is class imbalanced because human activities, by nature, have some activities of longer duration than others e.g., lying on bed is of longer duration than ambulating in the room; another reason is the availability of sensor readings within those activities due to the use of passive devices, as shown in Figure 5.

A recent study from our group introduced dynamically weighted CRF (dWCRF), a method that improves classification performance in class imbalanced data when training information is limited [43]. The classifier introduces a class related weight parameter into the objective function to give a higher cost to errors in minority classes. Therefore, given a training sequence ${\left\{x_{t}\right\}}_{t=1}^{T}$ associated with a label sequence ${\left\{y_{t}\right\}}_{t=1}^{T}$, where $y_{t}\in Y=\left\{1\cdots K\right\}$; the weighted log-likelihood function has the form

(2) $$L\left(\lambda ,w\right)=\sum_{t=1}^{T}w_{t}\log p\left(y_{t}|x_{t},\lambda \right)$$

where *λ* are the model parameters, $w_{t}$ are the dynamically calculated class related weight parameters that maximize the overall harmonic mean of recall and precision (F-score). Our dWCRF model evaluates in real-time the extracted features and produces marginal probabilities for each possible activity class. This approach, as opposed to our previous study in [28], takes into consideration the effects of class imbalance without adding complexity to the resulting model and is able to evaluate the occurrence of activities of interest in real-time. In our study, the dWCRF model was implemented based on the crfChain toolbox by Schmidt *et al.* [44]. Furthermore, obtaining an activity prediction model that maximizes recall and precision as opposed to accuracy is important because metrics based on the number of true negatives (*i.e.*, accuracy, specificity) do not give a fair metric in the case of imbalanced data, as true negatives for the minority class are the true positives of the other classes including the majority class, which usually dominates the prediction model.

In general, during activity prediction, a learned CRF model is used to classify complete series of observations described by their extracted features into a sequence of activities as in our previous study [28]. This approach cannot be used in our scenario where activity prediction is required in real-time as an alarm signal must be generated as soon as a bed or chair exit occurs. Therefore, we implemented a real-time activity prediction algorithm that produces the desired marginal probabilities for each class for each received observation by using the sum-product algorithm [45], and is described previously in [41]. The marginal probability inference has the form

(3) $$m_{k}\left(y_{k,t}|x_{t}\right){|}_{k=1}^{K}=\frac{1}{Z_{t}}\left(\exp\left(F\left(y_{t},x\right)\right)\sum_{y_{t-1}}\exp\left(F\left(y_{t-1},y_{t}\right)\right)m_{k}\left(y_{k,t-1}|x_{t-1}\right)\right)$$

Term $m(y_{t}|x_{t})$ are the predicted marginal probabilities for all *K* possible values of *y* given the observed sensor data $x_{t}$, $Z_{t}$ is a normalizing term and *F* is the potential function determined by feature functions from the data. In general, it is expected that inference of complete sequences will be more accurate than real-time inferencing; in the latter case, decisions are based on the current and past sensor observations, whereas, in a sequence, the inference process has disposition of past, current and future information about every observation in the sequence.

We consider that individual activity predictions (*i.e.*, activity with the highest marginal probability for each observation) are not of interest as raw noisy data is used and can induce single prediction errors resulting in emitting an unwanted alert to caregivers (Figure 1). Instead, in the activity recognition process, we use the marginal probabilities that represent the degree of confidence in each possible predicted activity for each sensor observation. Hence, marginal probabilities can be considered as normalized scores for each possible activity.

#### 3.2.3. Bed and Chair Exit Recognition

**Figure 6:**
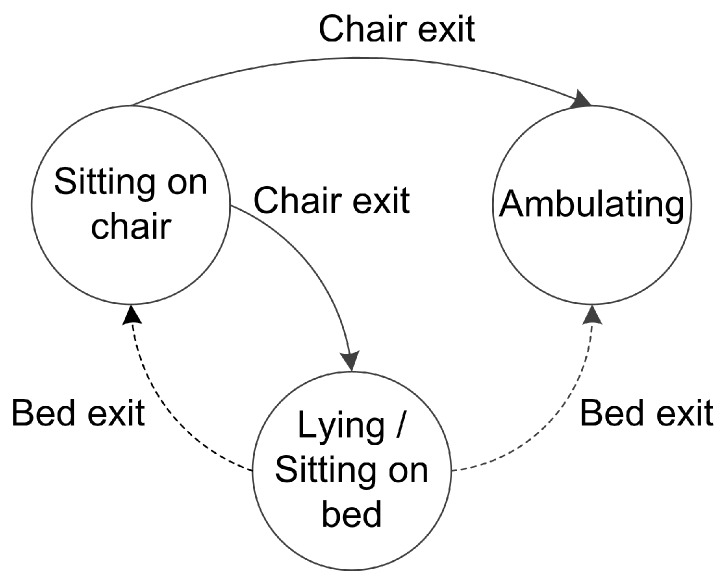
Bed and chair exit recognition. State machine transition model used to recognize a bed or chair exit.

In this stage, an alert signal is triggered on the occurrence of a recognized bed exit or chair exit as shown in Figure 6. To evaluate this occurrence, we propose a score function that first sums the normalized scores per activity over 1 s of data from the last processed observation (at time $t^{\prime }$) from the activity prediction stage. The assigned label $y_{t^{\prime }}$ is given by the expression:

(4) $$y_{t^{\prime }}=\underset{y_{k}}{arg max}\sum_{t=t^{\prime }-1\;s}^{t^{\prime }}m_{k}\left(y_{k,t}|X\right)$$

where $m_{k}$ is the marginal probability from the activity predictor stage, $y_{k,t}$ are all possible labels at time *t* and *X* are the observed sensor readings. The goal of the score function is to assign an activity class that is dominant in the 1 s of data and as a consequence is less affected by activity prediction errors caused by noise. We used a period of 1 s as we want to consider enough data to recognize a posture transition, but not exceed the minimal posture transition duration of 1.75 s, as periods longer than this can overlap valid posture transitions [27]. The score function then selects the activity with the highest sum and assigns that activity to the last processed sensor observation. This scoring function implements a noise removal process, filtering those erroneous predicted activities with high marginal probabilities that could potentially produce false alarms if unfiltered. In addition, performed activities are mostly represented by multiple predictions from multiple sensor observations, hence single predicted activities that are dissimilar to those predicted readings close in time are likely to be erroneous and noise induced. We compare the effectiveness of using our score function in Section 4.

The activity recognition process uses a simple finite state machine, as illustrated in Figure 6, and triggers an alert signal when either a bed or chair exit is identified in real-time by the activity recognition algorithm. Bed exit alerts are generated if an Ambulating or Sitting-on-chair predicted activity is preceded by either Lying or Sitting-on-bed. Similarly, Chair exit is identified if the previous predicted activity is Sitting-on-chair is followed by any other activity. We have included the recognition of bed exit when the participant transfers from bed to the chair and chair to bed for chair exit without ambulation, as it is possible to miss sensor observations while ambulating. After an alert is issued, we consider that it is physically impossible for an alert of the same type to occur in the next 1.75 s as this is the minimum time for a posture transition to take place; therefore we disregard any subsequent alert within the next 1.75 s period [27].

#### 3.2.4. Statistical Analysis

In this study, true positives (TP) were correctly recognized bed or chair exit alerts when: (i) the alert occurs when the person is actually performing an activity of interest; or (ii) the real activity (ground truth) occurs no more than a time T=5 s after the alert signal. False positives (FP) are recognized bed or chair exits that do not follow the TP criteria, *i.e.*, incorrectly identified as target activity. False negatives (FN) are bed and chair exits that were missed.

The performance of the system was evaluated using the metrics:

(5) $$\begin{array}{cc} \text{recall}\;(\text{sensitivity})= & TP/(TP+FN) \end{array}$$

(6) $$\begin{array}{cc} \text{precision}= & TP/(TP+FP) \end{array}$$

(7) $$\begin{array}{cc} \text{F-score}= & \left(2\times \text{recall}\times \text{precision}\right)/\left(\text{recall}+\text{precision}\right) \end{array}$$

We focus on these metrics as they consider the occurrence of errors in relation to TP. Given that we are focused on evaluating the occurrence of alarming activities, we do not focus on true negative metrics.

Evaluation of these metrics was performed using a 10-fold cross validation procedure that divides the data of a given dataset (*i.e.*, Room 1 dataset or Room 2 dataset) into 10 subsets, where six of these subsets were used for training (learning the dWCRF model in Figure 2), two subsets (validation set) for parameter selection and two subsets for testing which reports the test results of the model selected. In our case, each subset contains data from more than one participant; hence, it is possible that different trials of the same person are distributed in the training, testing and validation subsets, and, therefore, our results are not participant independent. However, use of 10-fold cross validation does allow us to obtain results that are less sensitive to the partitioning of the data. Best parameters were selected from the validation set of returned F-scores. Results are presented as mean ± standard deviation (STD).

For comparison of results, we use a two-tailed independent t-test; a *p*-value of *p* < 0.05 is considered statistically significant.

## 4. Results and Discussion

Fourteen healthy participants (average age of 74.6 ± 4.9 years) and male to female ratio of 2:5 participated in the study. In general, when using the score function, our method achieved a consistently higher F-score performance for all activities as shown in Table 1, with dWCRF [43] parameters *τ* (number of iterations) and *ϑ* (L2 regularization parameter) of values $\tau =8,\vartheta =3.1\times 10^{-4}$ for Room 1 and $\tau =1,\vartheta =3\times 10^{-4}$ for Room 2. As expected, the score function improves the overall F-score and precision (reducing false positives) at the expense of decreasing recall. This is because some short duration activities can be under-represented in a window and erroneously assigned to a more dominant class label in the window. Although there is overall improvement in mean performance metrics when using the score function, the differences are not statistically significant with *p* ≥ 0.14 for all metric comparisons; *p*-values not shown in Table 1.

These results show that room configuration Room 2 performs better for real-time bed exit recognition with statistical significance (*p* ≤ 0.001) as it obtains higher recall, *i.e.*, low missed bed and chair exits, and precision, *i.e.*, low false alarms, while having a more practical deployment than Room 1. In contrast, Room 1 achieved, in general, higher mean performance metrics for chair exits although only recall results were statistically significant (*p* < 0.001). These results are important as they indicate that a smaller deployment as that of Room 2, with two antennas facing the bed, achieves better results recognizing bed exits than a larger deployment as that of Room 1, with three antennas facing the bed.

We also present the results of our previous method in [28] where we evaluated a machine learning approach, specifically CRF, in contrast to the empirical approach in [27] to detect bed transfers. In [28], we only considered detection of bed exits, sequence prediction and a reduced number of classes and features. For this comparison, we detect chair exits and bed exits as illustrated in Figure 6, and we use the features, class labels and real-time inference algorithm used for this study. The results shown in Table 2, with CRF parameter ϑ=0.1 for both Room 1 and Room 2, indicate that our method achieves higher performance than the method in [28] for bed and chair exits in Room 2 and bed exits for Room 1; however, our method has a lower performance for chair exits in Room 1. Differences between F-scores are not statistically significant for all cases (*p* > 0.13) except for bed exits for Room 2 (*p* = 0.047), where our method significantly outperforms that of [28].

A fair comparison of our results with other studies is difficult as environmental settings and cohorts of people participating are not the same. In an earlier study, Capezuti *et al.* [46] used pressure sensors on beds to detect bed exits in nursing home residents; the tested system achieved recall metric of 71% and a low specificity of 0.3% [46]. This high false alarm rate might be one reason why pressure sensors have been found to be ineffective in recent clinical trials [7,8]. Other studies in the literature that focused on bed exits have reported recall and specificity values over 90%, but these studies were undertaken with young and middle aged adults which is a significant limitation [10,11].

**Figure 7:**
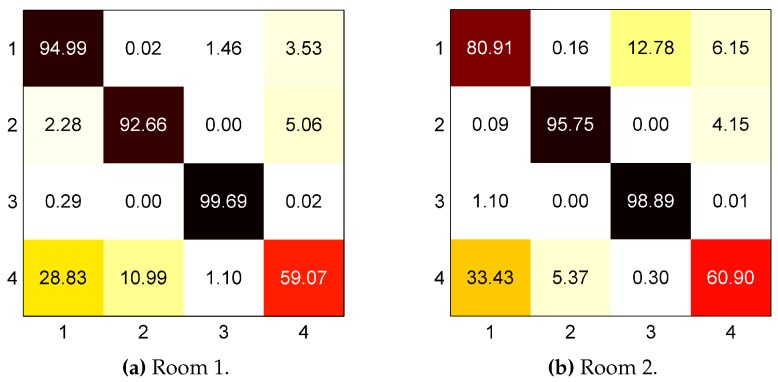
Confusion matrix for data of (**a**) Room 1 and (**b**) Room 2. Output of dynamically weighted conditional random field (dWCRF) classifier for labels 1: Sitting-on-bed, 2: Sitting-on-chair, 3: Lying and 4: Ambulating. Results in %.

There are various causes that affect the performance of the system given in Table 1 in both Room 1 and Room 2. Firstly, class imbalance due to the heterogeneous duration of activities, such as lying on bed and ambulating and the passive nature of our sensor affect the learned model of the classifier. Although we use a classifier that considers the effects of imbalance (dWCRF), the classifier is unable to completely discriminate minority class labels (especially Ambulating) as evidenced in the confusion matrix shown in Figure 7. Here, only about 60% of Ambulating sensor observations are correctly predicted. This problem resulted in the Ambulating class being incorrectly identified in approximately 30% and 5%–10% of cases as Sitting-on-bed, and Sitting-on-chair, respectively, see Figure 7. In both room configurations, Ambulating class is about 3.7% and 1.5% of total labels in Room 1 and Room 2, respectively, and correctly predicting these sensor readings as Ambulating is important to determine a bed or chair exit event as illustrated in Figure 6.

Secondly, the inadequate powering of the W^2^ISP and the random access nature of the air interface protocol EPC Class 1 Gen 2 [47] used by the RFID technology produces a variable number of readings per second [48], as evidenced in Figure 5. Inadequate powering maybe caused by incident RF power variations due to the sensor being occluded from the RFID reader antenna by the human body in different postures. Moreover, in [28], we demonstrated that placing the W^2^ISP at different distances and angles from the reader antenna affected the time required for the sensor to transmit data as a result of the variable levels of incident RF energy on the W^2^ISP. These causes aggravate the imbalance problem and also result in missing some posture transitions (sit-to-stand or stand-to-sit) or transitions being unobserved. Hence, the classifier has to discriminate between a person sitting and ambulating, where the trunk of the person is upright in both postures, and the failure of the current features to totally disassociate both postures lead to misclassifications. This is evident in the confusion matrix (Figure 7), where 3%–6% of readings for classes Sitting-on-chair and Sitting-on-bed were predicted as Ambulating. These errors caused false alerts (false positives) in Room 1, producing low precision (<60%) in bed and chair exit recognition. Room 2 is also affected, particularly the low performance of chair exits where classes Sitting-on-chair and Ambulating have the lowest amount of readings in the dataset (2.3% and 1.5% of the data, respectively).

Thirdly, at the end of some trials where the participant was required to exit the chair or bed and exit the room in Room 1, the participant exits the bed occluding the sensor with his body during the posture transition. In such occurrences, while walking a short distance towards the door, antenna1 and antenna4 facing the bed, fail to adequately energize the W^2^ISP and capture sensor readings. This caused misses (false negatives) for both chair and bed exits.

We also analyze the bed and chair exit recognition delays to understand the effects of using a passive sensor and our activity recognition method. The results in Table 3 show the average and median delay for recognized bed and chair exit events in relation to the ground truth data for both room settings. We can see that for both rooms the average delay is smaller than 3.3 s. We can also see that the average delays for bed exits are larger than those for chair exits. These longer delays are due to a lack of readings between the participant starting to ambulate after exiting the bed and sitting on the chair as the participant wearing the sensor is facing away from the RFID antennas. However, in general, the delay is low (median ≤1.25 s), especially for chair exits for Room 2 with median = 0. Absence of a delay, *i.e.*, delay =0 s, is possible when the last observation in the score function, corresponding to the first observation out of the chair, correctly determines an actual chair exit.

## 5. Conclusions

This technological development study for real-time activity monitoring to recognize bed and chair exits that incorporates a single RFID sensor worn by healthy older participants suggests promising preliminary results. In particular, Room 2 antenna positioning demonstrated that a smaller deployment designed to illuminate specific areas of interest can obtain significantly higher F-score (84%) for bed exits compared to that of Room 1 (59%), albeit a lower F-score (63%) for chair exits, although statistically not significantly different from that of Room 1 (77%). This is important in a practical context, as a reduction of antennas suggests a decrease of deployment cost for improved performance in bed exit recognition. This is because most of the cost corresponds to RFID infrastructure as RFID tags are inexpensive and maintenance free. Although RFID infrastructure costs can be relatively high compared to the cost of a sensor, it is important to note that RFID infrastructure is increasingly deployed in hospitals for applications such as patient and equipment tracking [34,35] and our proposed approach can easily leverage upon such existing infrastructure.

We have improved upon our previous study in [28] by enhancing the set of extracted features using a dynamically weighted classifier that considers class imbalance and produces real-time predictions of both bed exits and chair exits. The results of this initial study with older people to determine bed and chair exits in real-time are important due to the lack of methods in the literature that use wearable batteryless sensors for fall prevention on older people. In addition, the results are important, as they investigate the use of batteryless body worn sensors for activity recognition, especially with a cohort of older people.

Our study is not short of limitations, and thus further research must consider various areas of development for improvement. A system limitation, as evidenced in previous discussion, is the antennas’ positioning as it affects the collection of sensor observations. The lower chair exit performance for Room 2 indicates that further study is needed to secure better performance from chair exit events by improving antenna positioning strategies. Future studies should also consider placing the sensor on the shoulder to reduce occlusions from ceiling mounted antennas and evaluate its performance. Such an arrangement can potentially increase the data collected when the participant is sitting and ambulating, and thus contribute to increasing the performance of our approach as well as reducing delays to detect bed and chair exits.

Another area for future work is the development of inconspicuous sensors that are textile integrated, and smaller in size without losing performance [49]. In this direction, our group is using washable conductive textile material (RIPSTOP silver fabric) [50,51] to build the antenna of the sensor. Future work in the area of machine learning methods must investigate additional sources of information to extract robust features to help discriminate similar postures. Moreover, the short duration of the trials in this study may not represent the duration of a complete day at a hospital. Therefore, long term trials, including both day and night times, must be considered with a larger sample of hospitalized older participants to validate our approach and evaluate the acceptability of the device after wearing the sensor for long periods of time.

## Figures and Tables

**Table 1:** Bed and chair exit recognition results for two room configurations. Comparison of recognition of bed and chair exits with and without the use of a score function. *p*-value corresponds to comparison between Room 1 and Room 2 using score function.

		Without Score Function		Using Score Function		
		Room 1 (%)	Room 2 (%)	Room 1 (%)	Room 2 (%)	*p*-value (*p*)
Bed Exit	Recall	67.07±9.6	94.16±8.1	64.24±8.9	93.45±9.8	<0.001
	Precision	48.96±9.9	72.07±14.4	57.24±11.0	78.83±13.9	0.001
	F-score	55.98±8.2	80.50±8.7	59.77±7.7	84.4±7.9	<0.001
Chair Exit	Recall	95.98±7.0	61.75±22.5	94.87±7.2	60.50±21.2	<0.001
	Precision	61.05±17.7	67.92±27.4	67.02±16.6	70.74±22.6	0.68
	F-score	73.35±14.5	63.03±22.1	77.64±13.4	63.78±18.7	0.07

**Table 2:** Bed and chair exit recognition using previous method in [28].

		Room 1 (%)	Room 2 (%)
Bed Exit	Recall	72.64 ± 8.9	91.91 ± 9.7
	Precision	43.22 ± 8.7	66.93 ± 13.1
	F-score	53.96 ± 5.84	76.40 ± 8.8
Chair Exit	Recall	96.98 ± 4.9	61.75 ± 22.5
	Precision	71.13 ± 21.8	63.99 ± 30.1
	F-score	80.51 ± 16.14	60.55 ± 24.3

**Table 3:** Delay in recognition of bed and chair exit. Time in seconds.

		Room 1	Room 2
Bed Exit	Mean±STD	2.63 ± 4.08 s	3.22 ± 6.05 s
	Median	1.20 s	1.25 s
Chair Exit	Mean±STD	1.93 ± 2.55 s	2.15 ± 1.56 s
	Median	1.13 s	0.00 s
